# Coronavirus disease 2019 (COVID‐19) as a potential trigger for benign recurrent intrahepatic cholestasis

**DOI:** 10.1002/ccr3.5557

**Published:** 2022-03-10

**Authors:** Turan Çalhan, Elif Yivli

**Affiliations:** ^1^ Department of Gastroenterology Haseki Training and Research Hospital Istanbul Turkey

**Keywords:** benign recurrent intrahepatic cholestasis, coronavirus disease 2019, liver

## Abstract

Benign recurrent intrahepatic cholestasis (BRIC) is a rare disease characterized by recurrent severe itching and jaundice. Coronavirus disease 2019 (COVID‐19) is a multisystemic acute viral disease and the liver is frequently affected. Here, we wanted to present a BRIC case triggered by COVID‐19 infection, discussing it together with current information.

## Graphical abstract

We demonstrate a BRIC case triggered by COVID‐19 infection with typical patern of laboratuary tests and also normal magnetic resonance cholangiopancreatography imagine.

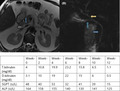

## 1 INTRODUCTION

Benign recurrent intrahepatic cholestasis (BRIC) is a rare autosomal recessive or sporadic disease characterized by recurrent severe itching and jaundice without significant liver damage. It was first described by Summerskill and Walshe in 1959.^1^ Although the disease can happen at any age, it usually starts at a young age and often occurs in the first decade. Disease attacks usually pass on their own after weeks and/or months without any damage.^2^ In spite of the fact that the main cause of the disease and the triggering factors that initiate the attacks are not fully known, genetic studies have found that there are defects in the ATP8B1 and ABCB11 genes.^3^, ^4^ Some people can have attacks many times a year, while others have them once in 10 years.^5^ Attacks typically begin with jaundice (conjugated hyperbilirubinemia), itching, weight loss, weakness, and malabsorption. Laboratory diagnostics can determine signs of cholestasis seen without signs of hepatocellular damage. Disease attacks are of similar character, and the severity and frequency of these attacks decrease with age.^6^

Despite the lack of data showing that viral infections can initiate an attack of BRIC, it is assumed that cholestatic attacks generally begin after an upper respiratory tract infection.^7^ Coronavirus disease 2019 (COVID‐19) is an acute viral disease caused by coronavirus, which led to a pandemic and to severe Acute Respiratory Syndrome Coronavirus‐2 (SARS‐CoV‐2).^8^ Although respiratory failure is the main cause of death, the disease has a systemic course and affects all organs, including the liver. The liver damage in COVID‐19 patients can be in the form of a direct effect of the infection caused by SARS‐CoV‐2 on the liver, an indirect effect of the systemic infection on the liver, hypoxic changes, the drugs used, or an acute exacerbation of an underlying liver disease.^8^ Post‐mortem studies in patients with COVID‐19 have shown bile duct proliferation, portal inflammation, and canalicular or ductular bile plugs in some patients..^9,10^ Here, we would like to present a BRIC attack triggered by COVID‐19 infection in a patient previously diagnosed with BRIC clinically, histopathologically, and genetically, and to discuss it in light of the current literature.

## 2 CASE PRESENTATION

**FIGURE 1:**
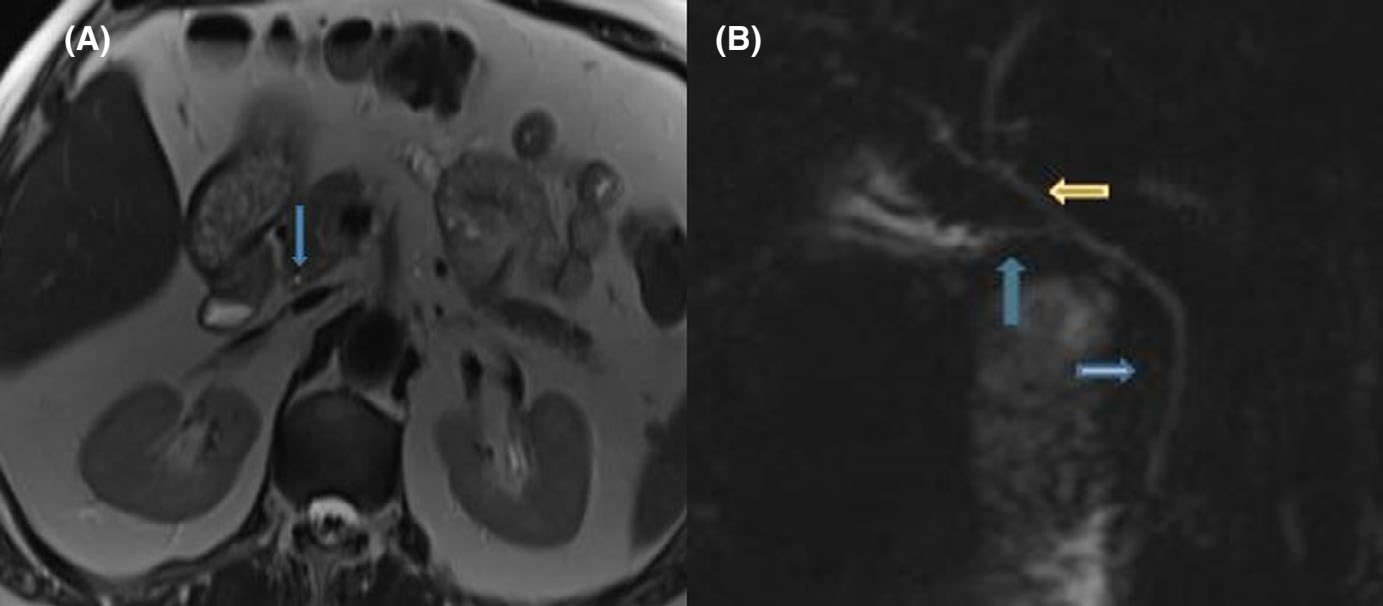
(A) axial magnetic resonance imaging (B) magnetic resonance cholangiopancreatography imaging. Extrahepatic and intrahepatic bile ducts appear normal in the patient. (Blue thin arrow: choledoch, yellow arrow: common bile duct, thick blue arrow: cystic duct)

A 59‐year‐old man was admitted to the emergency department with fever, cough, and shortness of breath that had been present and gradually increasing for 5–6 days. Fever was 37.8 C, arterial blood pressure was 120/70 mmHg, oxygen saturation in room air was 93%, and pulse was 92 beats/minute. The patient had a known diagnosis of BRIC for 20 years and had no other systemic diseases. On physical examination, the sclera were slightly sub‐icteric. Coarse crackles were heard in the bilateral middle and lower lobes of the lungs on auscultation. Heart sounds were normal. The abdomen was normal on palpation, without palpable organomegaly. Peripheral lymphadenomegaly was not detected. The patient was not on any medications. The patient had not recently taken alcohol or toxin preparations. Laboratory values at admission were as follows: white blood cell 3.11X103uL, hemoglobin 14 g/dL, platelet 84x103uL, C‐Reactive Protein 16 mg/L (normal range 0–5), procalcitonin 0.06 ng/mL, aspartate aminotransferase (AST) 78 U/L, alanine aminotransferase (ALT) 48 U/L, gamma glutamyl transpeptidase (GGT) 124 U/L, alkaline phosphatase (ALP) 164 U/L, lactate dehydrogenase 349 U/L, total bilirubin 4 mg/dl (normal <1.2), direct bilirubin 3.1 mg/dl (normal <0.3), INR:1, and albumin: 36 g/L. All other biochemical parameters such as urea, creatinine, electrolytes, amylase, and lipase were detected within the normal range. SARS‐CoV‐2 throat swab specimen (polymerase chain reaction‐PCR) collected from the patient due to the pandemic period tested positive. Chest computed tomography revealed a slight atypical viral pneumonia finding in both lungs. With the diagnosis of COVID‐19 infection, favipiravir, enoxaparin, nasal oxygen, and supportive treatment were initiated. At follow‐up, the patient's symptoms decreased, his respiration improved and his saturation returned to normal in a short time. However, direct weighted bilirubin levels continued to increase exponentially every week in the patient, whose initial direct bilirubin level was 3.1. Intrahepatic cholestasis was considered for the patient and examinations were performed for all possible causes of acute cholestasis; viral parameters, autoimmune hepatitis markers, hemochromatosis and screening for Wilson's disease, alpha‐1 antitrypsin level were all detected as normal. In magnetic resonance cholangiopancreatography, liver parenchyma was normal, and intrahepatic and extrahepatic bile ducts were normal (Figure 1A,B). While the COVID‐19 clinical symptoms regressed in the patient, the high bilirubin level that started with the COVID‐19 attack increased progressively and reached 22 mg/dl. In the table 1, the laboratory values of the patient are shown in 2 week periods. Severe itching and jaundice occurred in the patient. A diagnosis of BRIC attack was considered in the patient, since all the icteric causes were normal, the bilirubin pattern was compatible with BRIC, and the patient had a known diagnosis of BRIC. From the patient's medical history, we learned that he had similar attacks previously. Ursodeoxycholitic acid, anti‐histaminic, and cholestyramine treatment were started for BRIC. When there was insufficient response in the follow‐up, rifampicin treatment was added. With these treatments, bilirubin levels increased up to 22 mg/dl and peaked, then gradually decreased and returned to normal in 12 weeks. The patient had no permanent complications related to COVID‐19 and BRIC. The patient is on routine follow‐up without medication or complaints.

**TABLE 1:** Laboratory test results of the patient with two weeks of intervals are listed

	Week−0	Week−2	Week−4	Week−6	Week−8	Week−10	Week−12
T.bilirubin (mg/dl)	4	10.8	19.9	23.2	15.8	6.5	1.1
D.bilirubin (mg/dl)	3.1	10	19	22	15	6	0.5
SGPT (U/L)	48	40	53	32	41	39	15
ALP (U/L)	164	158	155	140	130	141	125

Note

T.bilirubin: total bilirubin, D.bilirubin: direct bilirubin, SGPT: serum glutamate–pyruvate transaminase, ALP: alkaline phosphatase.

## 3 DISCUSSION

Benign recurrent intrahepatic cholestasis (BRIC) is a rare disease that progresses with recurrent attacks of icterus and itching, and heals without significant liver damage. In BRIC patients who are completely asymptomatic between attacks, the attacks usually regress spontaneously and without treatment. The triggering factors that initiate the attacks are not fully known.^1^, ^11^ It has been published that stress, pregnancy, drugs (oral contraceptives), respiratory tract‐gastrointestinal, and cutaneous infections initiate the attack of BRIC.^2^, ^12^, ^13^, ^14^, ^15^ Halawi et al. suggested that a case of hyperthyroidism (Graves’ disease) initiated the disease attack in a patient with BRIC.^16^ When Zimmer et al. investigated the cause of prolonged hyperbilirubinemia in an acute hepatitis E virus (HEV) patient, they found a defect in the underlying ABCB11 gene, treated the patient as for BRIC (ursodeoxycholic acid) and claimed that the BRIC‐like episode was triggered by HEV.^17^ Blackmore et al. published a case in which lymphoma initiated an attack of intrahepatic cholestasis in a case diagnosed with Hodgkin's lymphoma, in which they detected ABCB11 and ATP8B1 genetic abnormalities.^18^ Although the trigger factor is not detected in most cases, these published cases indicate that BRIC attacks begin by interacting with some conditions. Therefore, we think that evaluating a patient with BRIC during an attack from this perspective can provide us with more information in the future.

We did not find any published data regarding the relationship between COVID‐19 and BRIC in the literature. Although the main involvement in COVID‐19 disease is in the lungs, it also affects the liver as a systemic infectious disease. Liver involvement has generally a slight course and manifests itself as increased transaminase and bilirubin levels. In the current pandemic, especially in severe disease, hepatic involvement is observed in 14%–53% of patients.^19^ Hepatic involvement may be caused by the direct cytopathic effect of the virus, uncontrolled immune response, sepsis, or medication. It has been detected that angiotensin converting enzyme 2 (ACE2) receptors, which are responsible for the entry of the virus into the cell and are often seen in type 2 alveolar cells, are also found in the gastrointestinal tract, vascular endothelium, and liver cholangiocytes.^19^ Chai et al. showed that in the liver, ACE2 receptors are more expressed in cholangiocytes (59.7%) than in hepatocytes (2.6%). ^20^ Interestingly, this information indicates that the liver is another potential target organ for SARS‐CoV‐2. Pathophysiologically, moderate adiposity, lobular and portal inflammation, apoptotic‐necrotic foci, and increased AST‐ALT in the laboratory can be observed generally in patients with COVID‐19. On the contrary, patients may also experience cholangiocellular damage in the form of bile duct damage‐proliferation, bile plugs, and laboratory elevation of ALP and GGT. Mutations in BRIC (ATP8B1 and ABCB11) cause cholestasis by disrupting the Bile Salt Export Pump (BSEP), which transports bile to the canaliculi.^21^ In BRIC patients, centrilobular cholestasis accompanied by bile accumulation in canaliculi, hepatocytes, and Kupffer cells is observed in liver histopathology examined during an attack.^2^, ^12^ On the one hand, the genetic tendency with a disorder in the transport of bile to the canaliculi in patients with BRIC, and on the other hand, the sensitive balance of the hepatocyte–cholangiocyte–canaliculi level being affected by external factors such as COVID‐19, may initiate an attack of the disease. Because the cholestatic pattern observed in COVID‐19 patients affects almost the same regions; however, this leads to a milder cholestasis condition. As we have shown in our case, in the presence of an underlying chronic liver disease (BRIC), cholestasis induced by COVID‐19 turns into a severe BRIC attack, and the BRIC attack can continue for weeks/months until the viral disease recovers.

There is no specific treatment for BRIC attacks. Anti‐histamines, cholestyramine, opioid antagonists, and ursodeoxycholic acid can be used for itching in mild/moderate cases. Rifampicin, plasmapheresis, and endoscopic nasobiliary drainage can be used in more severe cases to shorten both the symptoms and the duration of the attack.^22^, ^23^ A need for partial biliary diversion and liver transplantation is very rare.^24^, ^25^ In the current case, the attack lasted for 12 weeks and anti‐histaminic, ursodeoxycholic acid, cholestyramine, and rifampicin were used in the treatment.

In this case, we discussed that the fourth attack was triggered due to COVID‐19 infection in a patient who was diagnosed with BRIC genetically, histopathologically, and with laboratory tests, and who had three attacks so far. Before this diagnosis was established, the patient's history file had been examined in detail and the diagnosis of BRIC had been confirmed. Again, all possible causes of acute cholestasis were excluded by imaging and laboratory methods. Before his admission to the clinic, medication use that could initiate a possible attack or any toxic anamnesis were excluded, and no additional diseases were detected, except for COVID‐19 positivity. The elevation of bilirubin values before favipiravir treatment used for COVID‐19 suggested that it was unrelated to favipiravir. Favipiravir shows toxicity mostly in the form of liver enzyme elevations, and there are no data in the literature showing that it causes BRIC attacks. In this case, since clinical findings of the viral disease regressed within 1 week, and the direct bilirubin levels increased significantly every week, it was thought that there might be a BRIC attack triggered by COVID‐19 in the foreground.

In conclusion, as we hypothesized in the mechanisms above, SARS‐CoV‐2 shows a multisystemic course, and the liver is frequently affected in different conditions. In addition, it can lead to disease attacks in patients with underlying liver disease such as BRIC. This condition should be considered in the follow‐up of BRIC patient cases.

## CONFLICT OF INTEREST

The authors declare no conflict of interest.

## AUTHOR CONTRIBUTION

Turan CALHAN diagnosed the patient, followed up, wrote this case. Elif YİVLİ followed the patient, collected all data and helped to writing. All the authors contributed to the conduct of this work. All authors have read and approved the latest version of this manuscript.

## ETHICAL APPROVAL

None.

## CONSENT

Written informed consent was obtained from the patient to publish this report in accordance with the journal's patient consent policy (The patient is a 59‐year‐old male).

## Data Availability

The data is stored in the electronic database of the İstanbul haseki training and research hospital under the confidentiality act.
